# Strategies to Improve Bladder Control: A Preliminary Case Study

**DOI:** 10.3390/healthcare12181855

**Published:** 2024-09-15

**Authors:** Gesualdo M. Zucco, Elena Andretta, Thomas Hummel

**Affiliations:** 1 Department of Philosophy, Sociology, Education and Applied Psychology, University of Padova, 35131 Padova, Italy; 2 Centre for Mechanics of Biological Materials (CMBM), University of Padova, 35131 Padova, Italy; 3 Urology Unit, Spinal Unit ORAS, Motta di Livenza, 31045 Treviso, Italy; elenaandretta@libero.it; 4 Smell & Taste Clinic, Department of Otorhinolaryngology, University of Dresden, 01307 Dresden, Germany; thomas.hummel@tu-dresden.de

**Keywords:** low urinary tract symptoms (LUTSs), bladder residual, visual imagery, motor imagery, kinesthetic proprioceptor sensations, mental strategies, odor-based strategies, diuresis, associative learning, evaluative conditioning, case report

## Abstract

**Background:** Lower urinary tract symptoms (LUTSs) are a common complaint in adult and elderly men with bladder outlet obstruction, and have a considerable impact on their quality of life. Symptoms affect storage, voiding and post micturition stages. Among the latter, a feeling of incomplete emptying is one of the most bothersome for the patients; a condition that in turn contributes to affect urinary urgency, nocturia and frequency. Common recommendations include self-management practices (e.g., control of fluid intake, double-voiding and distraction techniques) to relieve patients’ symptoms, whose effectiveness, however, is under debate. **Methods:** In this report we describe two pioneering procedures to favor bladder residual content voiding in people complaining of LUTS disorders. The first is based on motor imagery and the second on the use of odors. The beneficial effects of Mental imagery techniques on various tasks (e.g., in the treatment of several pathological conditions or as valid mnemonics aids have a long tradition and have received consistently experimental support. Thus, a patient (a 68-year-old Caucasian man) complaining of LUTS was trained to use a motor imagery technique (building up a visual image comprising the bladder, the detrusor muscle and the urethra, and to imagine the detrusor muscle contracting and the flow of urine expelled) for 90 days and two odors (coffee and a lavender scented cleanser) for 10 days, as a trigger for micturition. He was asked to record—immediately after the first morning micturition—the time interval between the first (free) and the second (cued) micturition. **Results:** Reported data suggest the efficacy of motor imagery in favoring the bladder residual urine voiding in a few minutes (*M* = 4.75 min.) compared to the control condition, i.e., the baseline of the patient (*M* = 79.5 min.), while no differences between the odor-based procedures (*M* 1st odorant = 70.6 min.; *M* 2nd odorant = 71.1 min) and the latter were observed. **Conclusions:** A procedure based on an imagery technique may, therefore, be of general value—as a suggested protocol—and accordingly can be applicable to clinical settings. An olfactory bladder control hypothesis cannot, however, be ruled out and is discussed as a promising future line of research.

## 1. General Introduction

According to the European urology association (EAU) “lower urinary tract symptoms (LUTS)” are a common complaint in adult men with a major impact on quality of life and have a substantial economic burden” [1]. LUTSs are symptoms in the storage (e.g., increased frequency, urgency, nocturia) and voiding (e.g., slow stream, straining, intermittency) phase, as well as in post-micturition (e.g., feeling of incomplete emptying, dribbling) and can be related to obstruction during voiding or to bladder dysfunctions such as bladder over- or under-activity. Therefore, the management of male LUTSs is tailored according to the type of LUTS, considering underlying mechanisms and individual patient preference.

The therapeutic options range from herbal remedies to drugs (i.e., alpha blockers to relax the smooth muscle of the prostate, 5-alpha-reductase inhibitors to shrink the prostate, phosphodiesterase-5 inhibitors and antimuscarinics or beta-3 adrenoceptors to relax the bladder) and even surgical interventions.

However, in men with uncomplicated LUTSs, clinicians routinely suggest self-management. This includes lifestyle advice, such as control of fluid intake, moderation of intake of caffeine or alcohol, use of relaxed and double-voiding techniques, scheduled voiding, distraction techniques—such as mental tricks to take the mind off the bladder—and toilet and bladder retraining. Individualized intervention focusing on managing patients’ most bothersome symptoms are suitable, especially in storage LUTSs which have a greater impact on quality of life (e.g., on social functioning and sleep disturbance from nocturia). According to data collected through self-administered questionnaires, self-management guidelines show some evidence both in relieving patients’ symptoms and at slowing down progression of LUTSs [2,3,4,5].

Muscle relaxation and a double-voiding technique can also be recommended to decrease post-void residual, and Kegel exercises [6]—for pelvic floor muscle strengthening—can help to check unpredictable urinary urgency, time between voids and episodes of nocturia. Unfortunately, the effectiveness of these techniques is debatable and has not found experimental support [5].

At variance from the limits of self-management advice, the use of strategies based on mental imagery may represent a different viable and fruitful approach to help people complaining of LUTS disorders. The beneficial effects of mental imagery on various tasks have a long tradition and have received consistently experimental support. Such a strategy is the core of many mnemonic aids [7,8,9,10,11,12,13,14] as it has been shown to be valuable in the treatment of several pathological conditions. Indeed, the extant literature shows that psychiatric disorders (e.g., bipolar, post-traumatic stress disorders, anxiety disturbances, depression, phobias, obsessive and other personality disorders [8,15,16,17]) as well as neurologic diseases (e.g., Parkinson disease, motor impairments, strokes, unilateral neglect, neuropathic pains, traumatic brain injures [8,18,19,20,21,22]) greatly benefit from the use of mental imagery-based techniques, which favor rehabilitation and recovery. The same rationale applies in the treatment of physical disorders [23] and as a coadjuvant physical therapy [24]. The latter especially concerns sport players, easily at risk of injuries [25,26,27,28,29,30], whereas mental training practice is a fruitful tool to recover, improve physical skills and ameliorate performance. 

Mental imagery techniques can be experienced both from an internal and an external perspective. The first requires a person to focus on what is happening inside their body, namely that they are executing a mentally generated motor movement, accompanied by a proprioceptive/kinesthetic sensation in which their muscles are involved (i.e., are contracting or relaxing), thus mimicking what happens in an overt action. From an external perspective, a person is required to imagine seeing themselves while performing a motor act, as though they are an observer [31,32,33,34]. 

As visual mental imagery has been conceptualized as a depictive representation, analogous to a real perception [8], several studies have focused on its possible common neural substrate, as well as on the relationship between imagined and executed actions. Most available data—coming from neurophysiological records and structural and functional brain imaging studies—are consistent with this notion [8,31,35,36,37,38,39,40,41,42], showing activation in the same central brain regions; namely, the primary visual cortex and the pre-motor and parietal areas.

Based on the above well-documented literature on the efficacy of visual mental imagery and motor imagery in several domains, our main hypothesis aimed to verify whether this beneficial effect could be extended to urologic complaints. As outlined above, among post-micturition LUTSs, the sensation of incomplete emptying is a condition that in turn affects urinary urgency, nocturia and frequency; namely, the most bothersome LUTS for patients. Thus, the use of mental imagery strategies could be effective in favoring voiding the urinary bladder of its residual content, providing relief to the patients and the improvement of storage symptoms. 

A second pioneering hypothesis was to investigate whether the use of odors of beverages and foods expected either to favor diuresis or micturition (like, e.g., onion, coffee, artichoke, asparagus) or typically associated with the bathroom environment (like, e.g., a scented cleanser) could—if sniffed along with micturition—trigger the expulsion of residual urine content. The rationale of the hypothesis relies on the well-demonstrated role that odors play in several aspects of daily life, both in health (e.g., fostering the acquisition of early preferences through associative learning, governing the appreciation and aversion to foods, triggering old and vivid memories bearing emotional content [43,44,45,46,47]) and disease (e.g., acting as a biomarker—the sebum odor—revealing Parkinson’s disease or cancer biomarkers, as the presence of volatile organic compounds in urine, and furthermore as early markers of pathological conditions, signs of metabolic and infectious diseases or even indicators of fear [48,49,50,51,52,53,54,55,56,57]).

In this report we describe the case of a patient complaining of LUTSs, who was trained to use both motor imagery for a 90-day period, and two odorants (coffee and a lavender scented cleanser) for 10 days at a time, to investigate their effectiveness in promoting the decreasing of the post-void residual urine. We are not aware of other studies on this topic.

## 2. Methods

### 2.1. The Patient

G.A. was a 68-year-old man who complained of being afflicted with mixed LUTSs in the last 4 years. He reported having very bothersome symptoms such as urgency, frequency and nocturia 3–4 times per night and sensations of incomplete voiding. He filled out the International Prostate Symptom Score (IPSS, [58]), which evaluates the occurrence and severity of LUTSs; the questionnaire consists of 7 questions, 3 about storage symptoms, 4 about voiding symptoms and a question relating to quality of life (QoL). Each symptom is scored on a scale of 0–5 except the QoL question, which is scored from 0 to 6. Thus, the score can range from 0 to 35. A symptom score of 0–7 is considered mild, 8–19 is considered moderate and 20–35 is considered severe. G.A. reported a score of 13—moderately symptomatic—but expressed a great impact of storage LUTSs on his QoL when he answered “unhappy” to the question “If you were to spend the rest of your life with your urinary condition just the way it is now, how would you feel about that?”. The “8-Item Overactive Bladder questionnaire score” was also administered obtaining an abnormal score of 18 [59]. This questionnaire includes eight questions with answers ranging from zero (no symptoms or impact) to three (extreme symptoms or impact). The total score is the sum of all questions (minimum = 0, maximum = 24). An average micturition urgency of grade 2 according to the “Patient’s Perception of Intensity of Urgency Scale” (PPIUS) was detected. PPIUS [60] describes the degree of micturition urgency on a scale from 0 (no urgency) to 4 (urge urinary incontinence). 

Urine analysis and urine culture were negative, serum prostate-specific antigen was 9.8 ng/mL, physical examination and renal-bladder ultrasound results were unremarkable, with post-void residual urine of about 90 cc. Uroflowmetry revealed a mild obstructive flow pattern and multiparametric magnetic resonance imaging showed a large prostate (115 cc) without suspicious areas.

G.A. was treated with Serenoa repens with no benefit and then with tamsulosin with retrograde ejaculation disturbance and without efficacy on the LUTSs.

The test sessions occurred at the home of the patient. Informed written consent was obtained; the study was performed in accordance with the Declaration of Helsinki and the Good Clinical Practice of the European Community in the treatment of patients; the study was approved by the Bioethics Committee at the University of Padova (code number 740-a; 24 July 2024).

At the time of testing G.A. was in good health, fully alert and cooperative.

### 2.2. Materials and Procedures

The whole test procedure lasted 120 days, from September 2023 to December 2023, according to the following steps.

**Figure 1:**
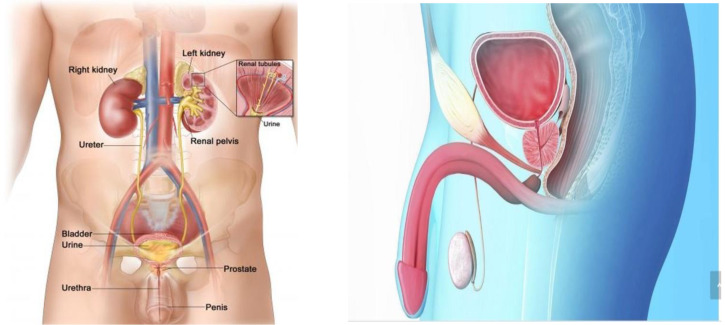
Examples of images used to train the patient in the use of motor imagery (picture on the left available from www.google.com/search?q=urinary+system, (accessed on 1 November 2023). National Cancer Institute (NCI); picture on the right, from the authors’ archive).

*Step 1 (motor imagery strategy acquisition)*: Firstly, an expert urologist provided the patient in simple words with information on the physiology of the urinary tract, focusing mainly on how the information run from the brain to the urinary tract when there is a need to empty the urinary bladder. Then, the patient was trained to build up a visual image comprising the bladder, the detrusor muscle and the urethra (see examples depicted in Figure 1). He was instructed to imagine a signal coming from the brain, running along the spinal cord and reaching the bladder. Then, to imagine the detrusor muscle contracting, the flow of urine coming out from the bladder, through the urethra and being expelled. He was asked to concentrate mainly on the act: contracting—expelling (and to rehearse mentally the motor task, which required about three days, according to his self-report), without any overt physical movement). When the patient was familiarized with the task, the first experimental session took place (Step 2).

*Step 2 (motor imagery strategy and bladder control)*: G.A. use the mental imagery strategy for three months according to the following rules. Every morning, immediately after breakfast, when in need of urination, the patient collected in a glass and weighted the expelled urine; immediately after that, he come back again to the bathroom and used the learned mental imagery strategy, waiting until the residual content of urine was expelled. This was again collected and weighted. The interval between the first (free) and the second (cued) micturition was recorded by means of a timer. During the task G.A. was suggested to close his eyes to improve concentration. The procedure was run meticulously by the patient, with the task performed between half past seven and half past eight in the morning of every day; the breakfast diet of G.A. for the whole period of testing did not vary (i.e., coffee, apple, and muesli with milk). 

*Step 3 (odor 1 strategy and bladder control):* According to the secondary hypothesis, G.A. was asked to associate to micturition an odorant expected to favor diuresis or micturition (since, as outlined in the introduction, some beverages and foods known to elicit diuresis have an odor). The selected odorant was “coffee”. The general procedure was almost the same as for the motor imagery experiment, with the difference that, during the second round (after the collection of urine from the first micturition) G.A. came back to the bathroom, he was requested to smell the odor of fresh coffee from a glass and to wait for a possible second micturition. According to the extant procedures, it was suggested to G.A. to sniff the odorant for about 4 s, with the stimulus kept approximately 2 cm in front of both nostrils. Intervals of a few seconds between sniffs were also suggested to avoid carry-over adaption effects. The waiting time between the first and the possible second micturition was fixed at about seven minutes. The odor 1 procedure was followed by G.A. for ten days. 

*Step 4 (odor 2 strategy and bladder control):* The general procedure here was the same as above (Step 3), with the difference that G.A. was asked to associate with micturition an odorant expected to be associated with a cleaned bathroom. We selected a natural and biological household cleanser with the odor of lavender (Gallo, Bio farm, Villanova di Camposampiero, Italy). The odor 2 procedure was followed by G.A. for ten days. 

*Step 5 (control condition, no intervention*): The general procedure here was the same as above, with the difference that GA was asked to not use any strategy while waiting for a possible second round micturition. The control procedure was followed for ten days. 

To summarize, the whole procedure followed by the patient was: 30 days motor imagery strategy; 10 days odor 1 strategy; 30 days motor imagery strategy; 10 days odor 2 strategy; 30 days motor imagery strategy; 10 days control condition.

## 3. Results

**Figure 2:**
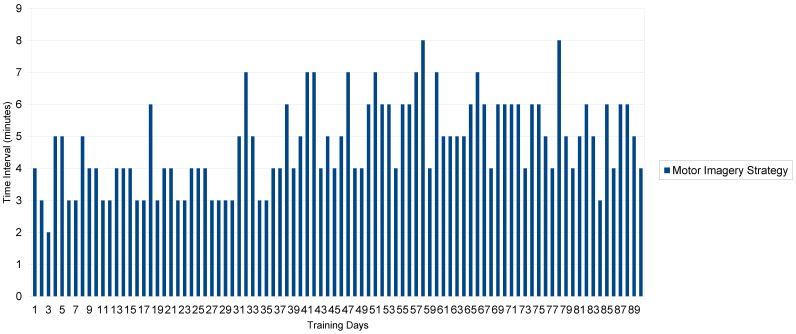
Time interval (minutes) between 1st and 2nd micturition in function of motor imagery strategy. (*M* = 4.75 ± 1.36 min), for a ninety-day period.

**Figure 3:**
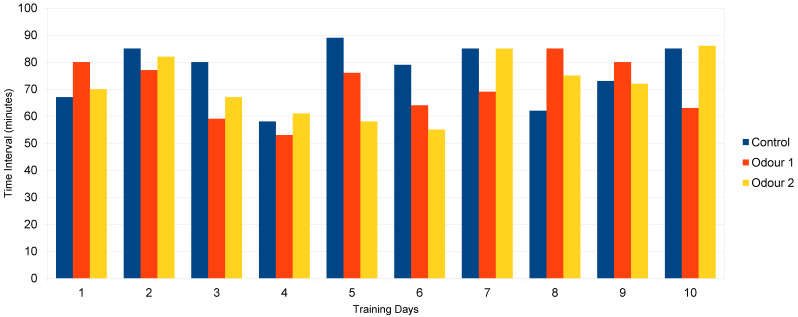
Time interval (minutes) between 1st and 2nd micturition in function of control (*M* = 79.5 ± 10.7). Odor 1 strategy (*M* = 70.6 ± 10.5) and odor 2 strategy (*M* = 71.1 ± 11.6) conditions for a ten-day period.

**Figure 4:**
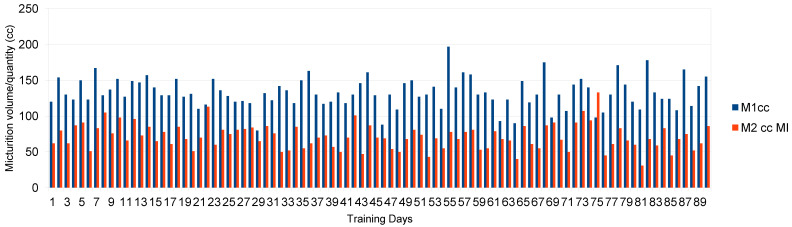
Expelled urine in first and second round (cc) in function of motor imagery strategy for a ninety-day period. Legend: M1cc: urine expelled at first micturition; M2ccMI: urine expelled at second micturition, post motor imagery.

Descriptive statistics are shown in Figure 2, Figure 3 and Figure 4. The patient’s mean scores (*M*) and standard deviations (*SD)* in the function of the four conditions were as follows:

*Motor imagery strategy:*

*M* expelled urine first round, cc 132. 7 ± 20.8; range: cc 80–197. 

*M* expelled urine second round, cc 71.3 ± 17.4; range: cc 31–133

*M* time interval between 1st and 2nd micturition, 4.75 ± 1.36 min.

Range: 2–7 min 

*Odor 1 strategy: M* time interval between 1st and 2nd micturition, 70.6 ± 10.5 min.

Range: 53–85 min

*Odor 2 strategy: M* time interval between 1st and 2nd micturition, 71.1 ± 11.6 (min).

Range: 55–86 min 

*Control condition: M* time interval between 1st and 2nd micturition, 79.5 ± 10.7 (min).

Range: 55–86 min

Post-training questionnaires administration showed as follows:

- IPSS decreased from 13 to 9, with improvement of voiding LUTSs, reduction in nocturia from 3 to 4 to 2 times per night and a 1-point improvement in QoL assessment.
- The “8-Item Overactive Bladder questionnaire score” decreased from 13 to 9.
- PPIUS remained unchanged at 2 (moderate urinary urgency).

These results suggest that the mental imagery strategy was successful in reducing bladder residual content voiding in a few minutes amount of time (see, Figure 2) at variance from the other conditions (see, Figure 3) whose scores are almost the same. 

Figure 4 provide a picture of the quantity (cc) of expelled urine in the few minutes between the first- and second-round micturition in the function of the motor imagery condition.

## 4. Discussion and Future Directions

In this study, we described a procedure to relieve the feeling of incomplete bladder emptying and to reduce the bladder post-void residual urine in a patient exhibiting storage LUTSs; a condition which is one of the most bothersome at post-micturition stage and that has a negative impact on urinary urgency, nocturia and frequency [3,4]. 

We proposed two training approaches, the first based on motor imagery and the second on the use of odors, based on the rationales of the effectiveness of mental imagery for various tasks [7,8,9,10,11,12,13,14,15,16,17,18,19,20,21,22,23,24,25,26,27,28,29,30,38] and the role that odors play in several aspects of daily life [43,44,45,46,47,48,49,50,51,52,53,54,55,56,57].

The results of the descriptive statistics unequivocally show that the short average time interval (*M* = 4.75 min) between the first and second micturition was due to the motor imagery strategy, at variance with the other conditions (*M* = 70.6 min for the odor 1 strategy; 71.1 min for the odor 2 strategy; and 79.5 min for the control, respectively) whose scores are almost the same and match the patient’ self-report about early-morning micturition. 

It is also worth stressing here that the level of satisfaction of the patient is high, and that he is currently successfully adhering to the strategy, both every morning when waking up and before going to sleep.

The motor imagery procedure shows, moreover, a further positive impact on storage LUTS disorders—i.e., urinary urgency, nocturia and frequency—since the possibility of patients voiding the bladder of all or most of its content plausibly lessens the frequency of micturition and also reduces the numbers of times patients wake up at night (when the motor imagery procedure is adopted before going to sleep; see the post-training questionnaire scores). This approach also limits the likelihood of bacterial bladder infections due to urinary residue [61]. Due to its feasibility, the strategy can be performed several times during the day and, in addition—if necessary—can be integrated with other powerful imagery cues; namely, imaging that the day is particularly cold, that the urgency is unstoppable and that it is necessary to quickly reach a place safe for micturition.

What about the use of odorants to manage LUTSs? Can the olfactory bladder control hypothesis be ruled out? We think not. Although not successful here, it could be a promising future line of research. Firstly, it is still plausible that an odor could trigger micturition if it has diuretic properties. Therefore, looking for one or more suitable odors is a challenge. 

Secondly, it is known that the evaluative conditioning paradigm (a form of associative learning) is a well-established and experimentally verified way to induce behaviors and emotions [62,63] and can represent here a good candidate for our purposes. In the present study, we assumed that the odor of a lavender-scented cleanser could have an associative relationship with the bathroom environment [64]; probably, however, this odorant was not so strictly linked this as to trigger micturition. Thus, according to the steps of the evaluative conditioning paradigm, a patient could be trained to learn an association between an odorant and micturition. Currently, we have a study underway to test this hypothesis, with the odor of lavender as the neutral stimulus (NS), to be sniffed before and during the proprioceptive sensation (i.e., the unconditioned stimulus, US) that accompanies micturition, and micturition as an unconditioned response (UR). According to the paradigm, following repeated association, the sole presentation of the odor (now, the conditioned stimulus, CS) should elicit micturition (now, the conditioned response, CR [63]).

Therefore, a suitable odor as a diuretic stimulus and bladder conditioning by means of the evaluative conditioning paradigm, can be successful strategies to help people to manage low urinary tract disorders. 

To conclude, the present study has demonstrated the usefulness of the motor imagery strategy in promoting the emptying of the bladder from the residual content of urine in a man suffering moderate storage LUTSs. We, therefore, recommend this procedure as a novel suggested protocol for clinical and experimental purposes. 

We acknowledge, however, that our study, has the limitation of being preliminary in nature and based on a case report. Accordingly, although single-subject designs are per se well-known and effective experimental procedures [65], further research, including large observational and randomized controlled trials, is needed.

## Data Availability

No data are available on the web due to privacy concerns.
